# Quencher-Free Fluorescence Method for the Detection of Mercury(II) Based on Polymerase-Aided Photoinduced Electron Transfer Strategy

**DOI:** 10.3390/s16111945

**Published:** 2016-11-18

**Authors:** Haisheng Liu, Linbin Ma, Changbei Ma, Junyan Du, Meilan Wang, Kemin Wang

**Affiliations:** 1 State Key Laboratory of Medical Genetics & School of Life Sciences, Central South University, Changsha 410013, China; 15675888046@163.com (H.L.); 3701130218@csu.edu.cn (J.D.); 2 Department of Environmental Monitoring, Changsha Environmental Protection Technology Vocational College, Changsha 410004, China; macblab@163.com (L.M.); cshbxyhxjys@163.com (M.W.); 3 State Key Laboratory of Chemo/Biosensing and Chemometrics, Hunan University, Changsha 410081, China; tnewstar@163.com

**Keywords:** quencher-free, Hg^2+^ ion, photoinduced electron transfer

## Abstract

A new quencher-free Hg^2+^ ion assay method was developed based on polymerase-assisted photoinduced electron transfer (PIET). In this approach, a probe is designed with a mercury ion recognition sequence (MRS) that is composed of two T-rich functional areas separated by a spacer of random bases at the 3′-end, and a sequence of stacked cytosines at the 5′-end, to which a fluorescein (FAM) is attached. Upon addition of Hg^2+^ ions into this sensing system, the MRS folds into a hairpin structure at the 3′-end with Hg^2+^-mediated base pairs. In the presence of DNA polymerase, it will catalyze the extension reaction, resulting in the formation of stacked guanines, which will instantly quench the fluorescence of FAM through PIET. Under optimal conditions, the limit of detection for Hg^2+^ ions was estimated to be 5 nM which is higher than the US Environmental Protection Agency (EPA) standard limit. In addition, no labeling with a quencher was requiring, and the present method is fairly simple, fast and low cost. It is expected that this cost-effective fluorescence method might hold considerable potential in the detection of Hg^2+^ ions in real biological and environmental samples.

## 1. Introduction

Heavy metals are a serious global problem because of their toxicity to the environment and human health [1,2]. Mercury(II) (Hg^2+^) is one of the toxic heavy metals on the list of the Agency for Toxic Substances and Disease Registry (ATSDR) of the US Department of Health and Human Services [3]. As a heavy metal, Hg^2+^ is easy to accumulate in human bodies but hard to biodegrade when it enters the body through food or water [4]. Numerous reports showed that Hg^2+^ can do serious harm to the kidney, brain, nervous systems and other organs, even at low concentrations [5,6,7]. According to the US Environmental Protection Agency (EPA) standard, the maximum allowable level for Hg^2+^ in drinking water is 10 nM [8]. Therefore, the development of a simple, sensitive, environmentally friendly, yet low-cost method for Hg^2+^ ions detection is needed [9,10].

Several traditional techniques have been used to detect Hg^2+^, such as atomic absorption/emission spectroscopy [11], cold vapor atomic absorption spectrometry (CVAAS) [12,13], X-ray absorption spectrometry [14] and inductively coupled plasma–mass spectroscopy (ICP-MS) [15]. However, those techniques are usually time-consuming, have poor specificity, are relatively costly and require large-scale instruments [16]. To overcome those obstacles, many types of Hg^2+^ sensors based on thymine-Hg^2+^-thymine (T-Hg^2+^-T) complexes have been invented, including fluorescence [16], colorimetric [17] and electrochemical sensors [18]. It is well known that Hg^2+^ can selectively bind thymine-thymine (T-T) in a sensitive and specific way to form the T-Hg^2+^-T complexes, so the biosenor based on T-Hg^2+^-T is usually specific, time-saving and highly sensitive [19,20]. Recently, singly-labeled smart probes, which take advantage of the selective quenching of fluorophores by neighboring guanosine residues via photo-induced electron transfer (PIET), were introduced [21,22,23,24]. They have been found to have wide applications in T4 polynucleotide kinase activity assay [21], DNA and RNA analysis [22,23], and lead ion detection [24]. In this paper, we report a novel simple and sensitive Hg^2+^ assay method using a smart probe based on polymerase-assisted PIET.

## 2. Materials and Methods

### 2.1. Reagents and Materials

Klenow fragment (KF-) polymerase (without 3′ to 5′ exonuclease activity) and 10x New England Biolabs (NEB) buffer 2 (100 mM Tris-HCl, 500 mM NaCl, 100 mM MgCl_2_, 10 mM DTT, pH = 7.9) were obtained from New England Biolabs (Beverly, MA, USA). DNA probe (5'-FAM-CCCCCCCCCGTT CTTCCCTTGTTCG-3') was synthesized by Sangon Biotechnology Co. Ltd. (Shanghai, China). dNTP mixture and TE buffer were purchased from Sangon Biological Engineering Technology and Services Co., Ltd. (Shanghai, China). The Mercury chloride (HgCl_2_) and silver nitrate (AgNO_3_) were purchased from Sigma Chemical Co. (St. Louis, MO, USA). The potassium chloride (KCl), lithium nitrate (LiNO_3_), calcium(II) nitrate tetrahydrate (Ca(NO_3_)_2_∙4H_2_O), copper(II) nitrate trihydrate [CuNO_3_)_2_∙3H_2_O], manganese(II) nitrate tetrahydrate [Mn(NO_3_) 4H_2_O], iron(III) nitrate nonahydrate (Fe(NO_3_)_3_∙9H_2_O), zinc chloride (ZnCl_2_) and iron(II) chloride tetrahydrate (FeCl_2_∙4H_2_O) were obtained from Sinopharm Chemical Reagent Co. (Shanghai, China). Ultra-pure water (18 MΩ∙cm^-1^, Kertone Ltd., Changsha, China) was used during all the experiments. The DNA probe was dissolved in TE buffer to 100 μM and stored at −20 °C for further use. 

### 2.2. Feasibility of the Strategy 

To demonstrate the feasibility of this assay, two samples were prepared: Sample A contains DNA probe, dNTP and KF polymerase, sample B contains DNA probe, dNTP, KF polymerase and Hg^2+^. The concentration of DNA probe, dNTP, KF polymerase and Hg^2+^ was 50 nM, 50 μM, 20 U/mL, and 500 nM, respectively. Each experiment was carried out in a final volume of 100 µL which containing 1x NEB buffer 2 (50 mM NaCl, 10 mM Tris-HCl, 10 mM MgCl_2_, 1 mM DTT, pH 7.9), and incubated at 37 °C for 5 min to a steady status, then Hg^2+^ was introduced into the solution, and the fluorescence measurements were carried out on an F2700 fluorescence spectrophotometer (Hitachi, Tokyo, Japan) with excitation at 490 nm and emission at 518 nm for FAM. The excitation slits and emission slits were set for 5.0 nm and 10.0 nm, respectively. 

### 2.3. Quantitative Analysis of Hg^2+^

To confirm the ability of the described strategy to sensitively detect Hg^2+^, with increasing concentrations of Hg^2+^ from 5 nM to 500 nM were added into the aqueous solutions and all other conditions were the optimized condition (50 nM, 100 dNTP and 20 U/mL KF polymerase.) Then, the sample was monitored by F-2700 to obtain the corresponding time curves. The rate of fluorescence quenching (Q%) was the signal in this experiment and the Q% could be calculated according to the follow equation:

(1) $$Q\%=\frac{F_{0}-F}{F_{0}}\times 100\%$$

F_0_ represents the fluorescence intensity before added Hg^2+^ while F stands for the fluorescence intensity after adding Hg^2+^ 400 s. 

## 3. Results and Discussion

### 3.1. Sensing Strategy

**Figure 1:**
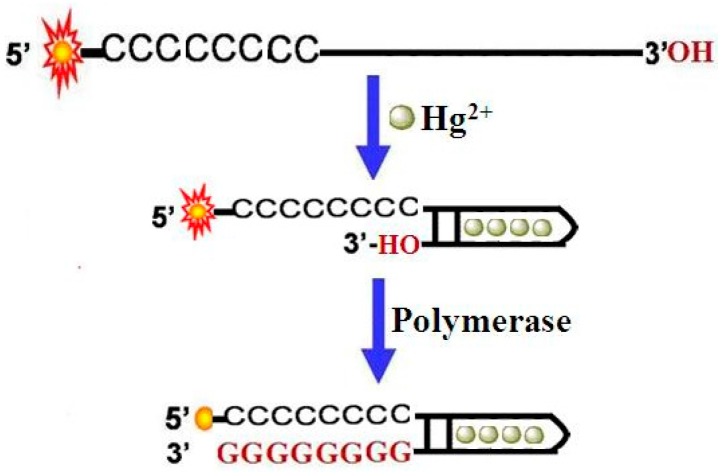
Schematic diagram of detection of Hg^2+^ based on polymerase-aided photoinduced electron transfer strategy.

A schematic illustration of the analysis of Hg^2+^ is shown in Figure 1. The DNA probe comprised of two domains was designed. The 3′-end is a mercury ion recognition sequence (MRS) that is composed of two T-rich functional areas separated by a spacer of random bases, and a sequence of stacked cytosines at the 5′-end, to which a fluorescein (FAM) is attached. Upon the addition of Hg^2+^ ions into this sensing system, the MRS folds into a hairpin structure at the 3′-end with Hg^2+^-mediated base pairs. In the presence of DNA polymerase, it will bind to the 3′-end of the probe and catalyze the extension reaction, resulting in the formation of stacked guanines, which will instantly quench the fluorescence of FAM through PIET. According to the fluorescence signal change of the detection system, Hg^2+^ can be detected easily.

### 3.2. The Feasibility of the Assay

**Figure 2:**
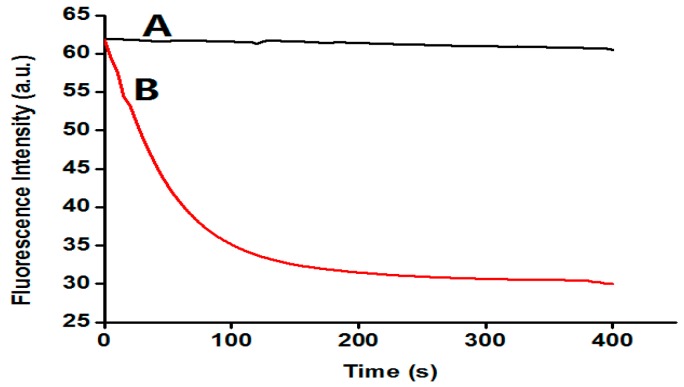
Fluorescence spectra of this sensing system in the absence (**A**) and presence (**B**) of 500 nM Hg^2+^ ions (50 nM DNA probe, 50 μM dNTP, 20 U/mL KF polymerase).

A set of experiments was carried out to verify the feasibility of our proposed strategy. The fluorescence intensity of these samples was monitored and the time courses were plotted in Figure 2. As illustrated in Figure 2, after the addition of Hg^2+^, there were no fluorescence changes in curve A (corresponding to samples A). This is as expected: in the absence of Hg^2+^, the MRS cannot fold into a hairpin structure, and the polymerase-catalyzed primer extension reaction is not triggered. In sample B, represented by curve B, after the addition of Hg^2+^, the fluorescence was decreased rapidly. According to the mechanism demonstrated in Figure 2, the extension reaction took place, and resulted in the formation of stacked guanines, which will instantly quench the fluorescence of FAM through PIET. In addition, the control experiment showed that Hg^2+^ at a concentration of less than 500 nM had no influence on the fluorescence of the DNA probe itself (data not shown). Therefore, a simple but effective strategy for the Hg^2+^ ion assay can be established.

### 3.3. Optimization of the Reaction Conditions

**Figure 3:**
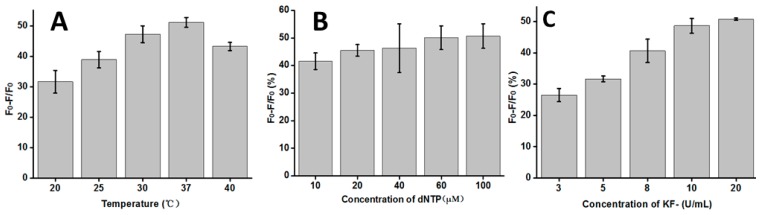
Optimization of the reaction conditions: (**A**) temperature (50 nM DNA probe, 50 μM dNTP, 20 U/mL KF polymerase); (**B**) concentration of dNTP (50 nM DNA probe and 20 U/mL KF polymerase); (**C**) concentration of KF polymerase (50 nM DNA probe and 60 μM dNTP). Error bars show the standard deviation of three experiments.

To achieve a better sensing performance, three main influencing factors were optimized through a series of experiments, including the temperature, and concentrations of dNTP and KF polymerase. The temperature was a crucial parameter for the T-Hg^2+^-T mismatched structure formed and the activity of the KF polymerase. As observed in Figure 3A, the rate of fluorescence quenching (Q%) increased with the increasing temperature, and then reached its maximum at 37 °C. When the temperature was higher than 37 °C, though, the rate of fluorescence quenching decreased again. This observation may explain the fact that the higher temperature will decrease the activity of the KF polymerase. Therefore, the temperature of 37 °C has been chosen as the optimum temperature in the following experiments. The concentration of dNTPs and KF can influence the extension reaction efficiency, and then block our reaction. As shown in Figure 3B, with the dNTP concentration increasing, the rate of fluorescence quenching gradually increased, and then reached equilibrium after 60 μM. Thus, 60 μM would be used as the optimum concentration of dNTP in the following experiments. Figure 3C shows the changes of the rate of fluorescence quenching with the concentration of the KF polymerase. It was observed that the rate of fluorescence quenching firstly increased and then reached a stabilized plateau when the concentration of KF polymerase was higher than 10 U/mL. In order to obtain highly effective replication, 20 U/mL of KF polymerase was used in the following experiments. In summary, the optimum reaction conditions were 37 °C, 60 μM dNTP, and 20 U/mL KF polymerase.

### 3.4. Quantitative Analysis of Hg^2+^

**Figure 4:**
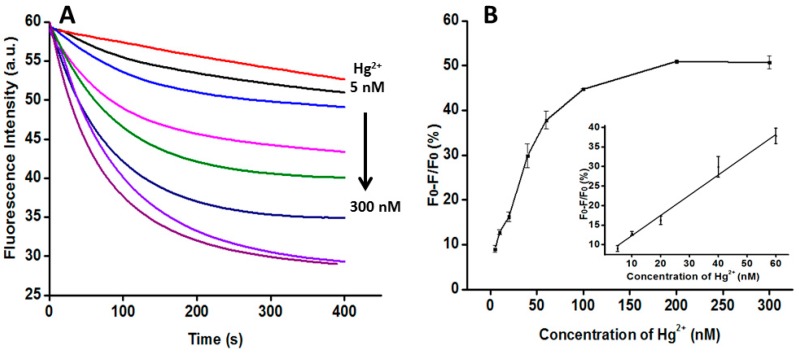
Quantitative analysis of Hg^2+^. (**A**) The time curves for the guanine base quenching sensor by different amounts of Hg^2+^ (5 nM, 10 nM, 20 nM, 40 nM, 60 nM, 100 nM, 200 nM, and 300 nM). The concentrations of DNA probe, dNTP and KF polymerase were 50 nM, 60 μM and 20 U/mL, respectively; (**B**) The linear relationship between the rate of fluorescence quenching (Q%) and the concentration of Hg^2+^. Inset: the linear relationship at low Hg^2+^ concentration. Error bars show the standard deviation of three experiments.

The concentrations of Hg^2+^ from 5 nM to 300 nM (5 nM, 10 nM, 20 nM, 40 nM, 60 nM, 100 nM, 200 nM, and 300 nM) were added to the solution and the corresponding fluorescence change was detected by F-2700. Figure 4A shows the time course of real-time monitoring of the guanine base quenching sensor at the excitation wavelength of 490 nm and the emission wavelength of 518 nm by different amounts of Hg^2+^. The fluorescence quenching increased as the concentration of Hg^2+^ increased. Figure 4B shows the linear relationship between the rate of fluorescence quenching (Q%) and the concentration of Hg^2+^. In Figure 4B, X is the concentration of Hg^2+^, and Y is the rate of fluorescence quenching. The regression equation is Y = 0.0517X + 7.154 with the concentration of Hg^2+^ ranging from 5 nM to 60 nM (R^2^ = 0.977). The limit of detection was estimated to be 5 nM, lower than the maximum allowable level for Hg^2+^ in drinking water according to the US EPA standard [8].

### 3.5. The Selectivity of this Assay

**Figure 5:**
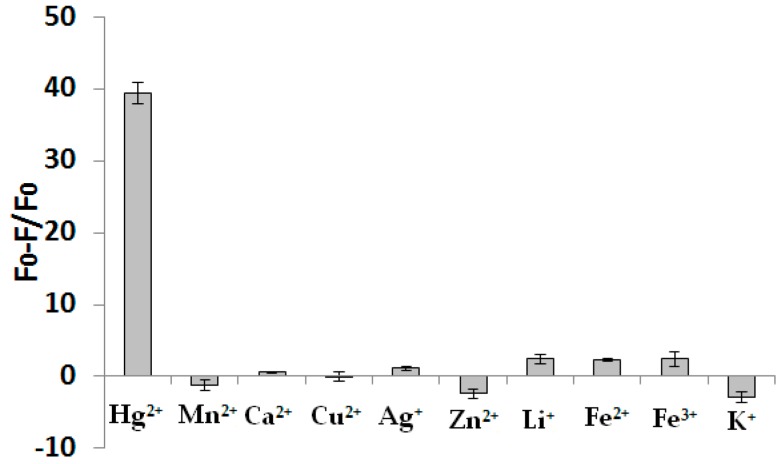
Selectivity of the analysis of Hg^2+^ by our method. The concentration of Hg^2+^ was 100 nM, and the concentrations of Mn^2+^, Ag^+^, Ca^2+^, Cu^2+^, Zn^2+^, Li^+^, Fe^2+^ and Fe^3+^ were kept at 2 μM and the concentration of K^+^ was 20 mM (50 nM DNA probe, 60 μM dNTP, 20 U/mL KF polymerase). Error bars show the standard deviation of three experiments.

To investigate the selectivity of this novel fluorometric method for Hg^2+^ detection, a number of environmentally relevant metal ions including Mn^2+^, Ag^+^, Ca^2+^, Cu^2+^, K^+^, Zn^2+^, Li^+^, Fe^2+^ and Fe^3+^ were tested. The concentrations of Mn^2+^, Ag^+^, Ca^2+^, Cu^2+^, Zn^2+^, Li^+^, Fe^2+^ and Fe^3+^ were kept at 2 μM and the concentration of K^+^ was 20 mM. As shown in Figure 5, none of these metal ions could induce the distinct decrease in fluorescence, except Hg^2+^ (100 nM). These results clearly demonstrate that the sensor, which is based on the ability of Hg^2+^ to form the stable structure of the T-Hg^2+^-T complex, is highly specific.

### 3.6. Detection of Hg^2+^ in Real Water Samples

We obtained the real water samples from the Xiang-jiang River in Changsha, Hunan Province. Then different concentrations of Hg^2+^ (20 nM, 60 nM, 100 nM) were added to the river water sample. As shown in Table 1, the analytical recovery of the added Hg^2+^ with our method was estimated in the range of 90.50%–103.10%, which demonstrated that this assay was reliable for the detection of Hg^2+^ in real samples.

## 4. Conclusions

In summary, we have developed a quencher-free fluorescent biosensor for the detection of Hg^2+^ based on polymerase-aided PIET. Under optimal conditions, the limit of detection for Hg^2+^ ions was estimated to be 5 nM which is higher than the US EPA standard limit (10 nM). In addition, the DNA probe containing the T-T mismatch pair selectively binds the Hg^2+^ ions as an acceptor, and thus this method has excellent selectivity towards other metal ions. Furthermore, no labeling with a quencher was required, and the present method is fairly simple, fast and cost-effective. Therefore, this method might hold considerable potential for the application of Hg^2+^ ion screening from a wide range of biological and environmental samples.

## Figures and Tables

**Table 1:** Detection of Hg^2+^ in real water samples (50 nM DNA probe, 60 μM dNTP, 20 U/mL KF polymerase).

River Sample	Added (nM)	Mean Found ^a^ (nM)	Mean Recovery (%)
1	20.00	18.10 ± 0.50	90.50
2	60.00	59.80 ± 1.80	99.80
3	100.00	103.10 ± 2.75	103.10

^a^ Mean of three measurements ± standard deviation.
